# The Optimization Study of Rheological Characteristics of Wind Power Grease Based on Gel-State

**DOI:** 10.3390/gels10040253

**Published:** 2024-04-09

**Authors:** Han Peng, Defang Zhao, Linjian Shangguan, Songyin Li, Ruixue Cheng, Yanchi Li

**Affiliations:** 1 School of Mechanical Engineering, North China University of Water Resources and Electric Power, Zhengzhou 450045, China; 15225515138@163.com (D.Z.); songyin1998@163.com (S.L.); 18741466255@163.com (Y.L.); 2 School of Computer Engineering and Digital Technology, Teesside University, Middlesbrough TS1 3BA, UK; r.cheng@tees.ac.uk

**Keywords:** wind turbines, the gel-state grease, carbon emissions, rheological characteristics

## Abstract

The gel-state grease plays a vital and indispensable role in the long-term operation of wind turbines. To reduce carbon emissions and increase the reliability of wind turbines, this paper takes the gel-state Mobil SHC 461WT grease as the study object. Firstly, the rheological properties of the gel-state Mobil SHC 461WT grease were investigated using the Anton Paar MCR302 rotational rheometer. Secondly, the rheological characteristics of three different gel states of the Mobil SHC 461WT grease (additive content of 0.1% of RFM3000, SK3115, and PV611, respectively, in the gel-state Mobil SHC 461WT grease) were optimized under the same conditions. Finally, according to the experimental results and the Herschel–Bulkley (H–B) model, the RFM3000 additive has the best effect on improving the rheological characteristics of the gel-state Mobil SHC 461WT grease. This research provides a new idea and direction for the technological advancement of the gel-state grease industry.

## 1. Introduction

With the rapid development of the global economy and the acceleration of the industrialization process, the issue of carbon emissions has increasingly attracted attention and focus [1,2]. Carbon emissions not only have a significant impact on climate change but also pose a great threat to human survival [3,4]. In this case, how to reduce carbon emissions has become one of the most important issues to be solved in today’s society [5,6]. To deal with this challenge, wind power has been used as one of the most important tools for the rational exploitation of wind energy [7]. It not only helps to alleviate economic problems but also significantly improves the availability of electricity [8,9]. However, friction wear is an unavoidable phenomenon in wind turbines [10]. To reduce energy consumption and extend the service lifespan of wind turbines, grease plays a dual role with its excellent rheological characteristics, which can effectively lubricate and provide excellent sealing effectiveness [11,12]. However, the carbon emissions generated during the preparation and utilization of traditional grease have become an important factor limiting the sustainable development of wind turbines [13,14]. The choice of using gel-state grease for wind turbine components has many advantages over regular grease or liquid lubricants [15]. Firstly, the gel-state grease is more viscous and can better adhere to the surface of the parts to form a long-lasting protective membrane, effectively reducing friction and wear [16]. Secondly, the consistency and viscosity of the gel-state grease are more suitable for application in the high-speed operating environment of wind turbines, providing stable and persistent lubrication [17]. Moreover, the gel-state grease also has better high-temperature resistance and oxidation resistance, which can maintain a stable lubrication effect in harsh operation environments, which extends the service lifetime of the parts and improves the reliability of the equipment [18]. Meanwhile, the additives (MoS_2_, TiO_2_, Al_2_O_3_, etc.) play a very important role in the rheological properties of gel-state grease [19]. They have the function of improving the lubrication performance of gel-state grease, enhancing the efficiency and lifespan of wind power generation equipment, and reducing maintenance costs, thus effectively promoting the development of related industries. However, if used inappropriately, the gel-state grease may lead to pollution of the environment, adversely affecting ecosystems and harming biodiversity and ecological balance. Therefore, when using gel-state grease additives, it is necessary to comprehensively consider the economic benefits and environmental impacts and take appropriate management measures to ensure a balance between economic development and ecological protection [20,21].

To optimize the lubricating characteristics of grease, many researchers have optimized the additives composition of grease. Mousavi et al. [22] studied the tribological and thermophysical behavior of ZnO nanoparticles in diesel fuel, using them as additives. They found that the addition of ZnO nanoparticles to diesel fuel significantly improved its tribological characteristics. When 0.7 wt% of ZnO nanoparticles were added to the diesel fuel, the pour point decreased by about 15% and the flash point increased by about 6%. Mousavi et al. [23] used MoS_2_ as an additive for diesel fuel and found by friction and wear tests that the addition of MoS_2_ did not have a significant effect on the anti-wear characteristics of diesel fuel. Xiong et al. [24] used fluorinated graphene as the polyurea grease additive. They found that fluorinated graphite and fluorinated graphene had a significant effect on the taper in, stencil oil separation and evaporation loss of polyurea grease by a friction and wear tester. Liang et al. [25] used calcium carbonate nanoparticles as fillers in the gel grease. They found that the filler can effectively improve the anti-wear and friction reduction performance of grease and reduce the use of grease raw materials, which is beneficial to the environment. Xu et al. [26] prepared and experimented with various gel-state greases with different molar ratios of hydroxystearic acid and lithium hydroxide monohydrate as thickening agent raw materials. They experimentally concluded that hydroxystearic acid at 10% acts as a structural improvement for lithium-based greases. Dai et al. [27] added 0.5% methyl oleate to weak gel drilling fluids in order to improve the application of ester lubricants in weak gel drilling fluids. They found through friction and wear tests that the lubrication factor of the drilling fluid was reduced by 95.65%. Jopen et al. [28] compared the formation of polyester organogels in three different base oils (Brightstock 150, Spectrasyn 40, and ricinoleic acid triglyceride) after rheological analysis and other methods. They found significant structural differences in the interaction of polymers as well as polyesters with their respective base oils. Schwack et al. [29] tested six industrial fats and oils with different compositions. They concluded experimentally that the grease lubricant with a low base oil viscosity and high bleed rate had the best anti-wear characteristics. Ng et al. [30] experimentally concluded that graphene nanoparticles in high oleic acid can significantly reduce the friction and wear of bearings. Saidi et al. [31] prepared MoS_2_ nanolubricant additives. They demonstrated the additive’s high anti-wear and friction-reducing capabilities through tribometer and wear tests. Wu et al. [32] used hexagonal boron nitride and calcium carbonate nanoparticles as base oil additives, which effectively suppressed the vibration generated during bearing operation. Li et al. [33] developed a gel-state grease for wind turbine generator bearings. Compared with imported grease, their development of gel-state grease meets or exceeds the level of imported products in terms of performance. Rosenkranz et al. [34] produced four different gel-state greases based on mineral-based oils by varying the amounts of thickeners and ZDDP additives. Pinilla-Peñalver et al. [35] proposed a new method for synthesizing polyurethane-based aerogels by using different levels of solids, organic solvents, and moisture. Table 1 summarizes the progress made by researchers on additives in lubricants.

To reduce carbon emissions and increase the efficiency and lifetime of wind energy generation systems, in this paper, the gel-state Mobil SHC 461WT grease is used as the study object. Firstly, the lubricant properties of gel-state Mobil SHC 461WT grease were evaluated by using an Anton Paar MCR302 rotational rheometer. Secondly, the rheological characteristics of three different single-additive (RFM3000, SK3115, and PV611) gel-state Mobil SHC 461WT greases were tested under the same conditions to analyze the effect of different additives on the rheological characteristics of gel-state greases. Finally, by analyzing the experimental results and the Herschel–Bulkley (H–B) model, we wish to find the additives that have the best effect on improving the rheological characteristics of the gel-state grease in order to provide theoretical guidance and technical support for the research of gel-state lubricating grease in the field of wind power generation and to contribute to the construction of low-carbon and high-efficiency wind power generation system.

## 2. Results and Discussion

### 2.1. Effect of Different Additives on the Rheological Characteristics of the Gel-State Mobil SHC 461WT Grease

#### 2.1.1. Lubrication Characterization of the Gel-State Mobil SHC 461WT Grease

**Figure 1:**
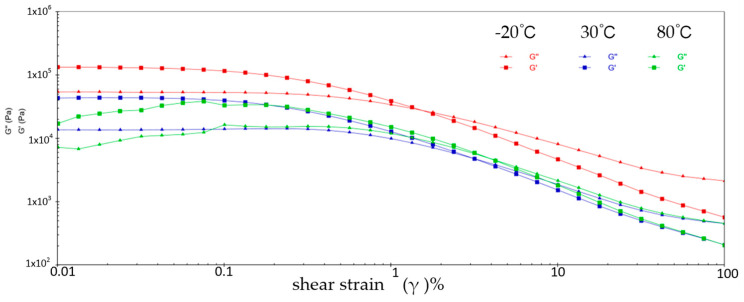
The viscoelastic modulus–shear strain curve for sample 1.

Figure 1 shows the variation patterns of viscoelastic modulus versus shear strain for sample 1 at three different temperatures. It can be learned that the energy storage modulus (G′) and loss modulus (G″) of sample 1 decrease with increasing shear strain at three different temperatures. This is because at low shear strains, the molecular chains and structure of sample 1 are still relatively ordered and the viscoelastic modulus is relatively high. With the increase of shear strain, the molecular chain and structure of sample 1 are subjected to greater perturbation and deformation, and the molecular structure is gradually destroyed, resulting from the decrease of the viscoelastic modulus of sample 1. During the reduction of G′ and G″, the two intersect when the shear strain reaches a certain value, which is called the flow point. Sample 1 is in a gel state before the flow point when G′ > G″. With the appearance of the flow point, i.e., G′ ≤ G″, sample 1 exhibits a flow state, which signifies a shift from elastic deformation to predominantly viscous deformation. At −20 °C, the flow point strain value for sample 1 is 1.538% and G′ = G″ = 2.7644 × 10^4^ Pa; at 30 °C, the flow point strain value of sample 1 is 3.118% and G′ = G″ = 4.831 × 10^3^ Pa; at 80 °C, the flow point strain value for sample 1 is 3.826% and G′ = G″ = 4.892 × 10^3^ Pa. In addition, the viscoelastic modulus of sample 1 shows a significant fluctuation phenomenon at 80 °C, indicating the instability of sample 1 in the high-temperature environment.

**Figure 2:**
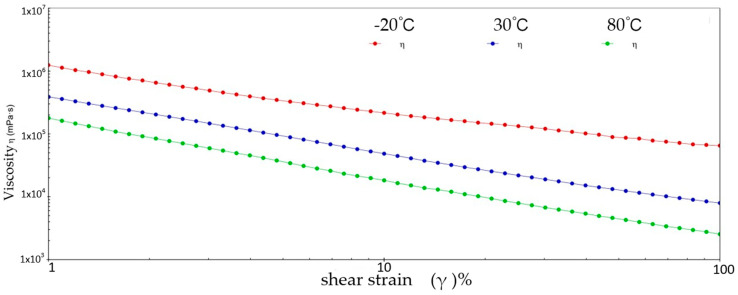
The viscosity–shear rate curve for sample 1.

**Figure 3:**
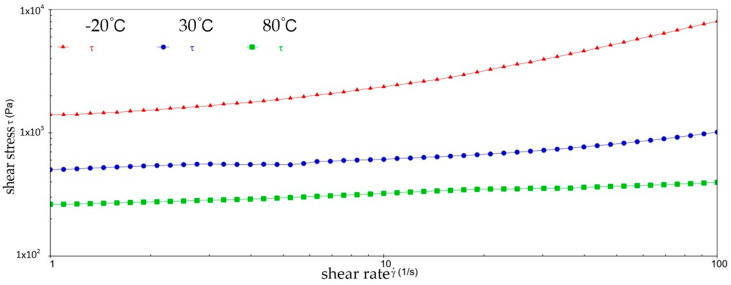
The shear stress–shear rate curve for sample 1.

Figure 2 and Figure 3 show the viscosity–shear rate and shear stress–shear rate curves for sample 1. It can be learned that at three different temperatures, the viscosity of sample 1 increases with increasing shear rate and the shear stress decreases with increasing shear rate, and all of them are linear. At −20 °C, the viscosity of sample 1 decreased from 1.41 × 10^6^ mPa·s to 8.01 × 10^5^ mPa·s, and the shear stress increased from 1.4041 × 10^3^ Pa to 8.0126 × 10^3^ Pa; at 30 °C, the viscosity of sample 1 decreased from 5.03 × 10^5^ mPa·s to 1.01 × 10^4^ mPa·s and the shear stress increased from 5.028 × 10^2^ Pa to 1.0128 × 10^3^ Pa; at 80 °C, the viscosity of sample 1 decreased from 2.63 × 10^5^ mPa·s to 3.96 × 10^3^ mPa·s and the shear stress increased from 2.6283 × 10^2^ Pa to 3.962 × 10^2^ Pa. At −20 °C, the viscosity decreases slower with increasing shear rate, indicating that sample 1 has high viscosity and strong adhesion at low temperatures. This is due to the low temperatures causing sample 1 to become more viscous and difficult to flow or release under shear. However, at 30 °C, the rate of decrease in viscosity of sample 1 is excessive, which reflects the presence of shear dilution of sample 1. The faster shear dilution characteristics may cause sample 1 to lose viscosity at high shear rates, which prevents the formation of an effective lubrication film and reduces lubrication effectiveness. In addition, excessive shear dilution can lead to rupture of the lubricant film or loss of grease, negatively affecting the long-term stability of the gel-state grease.

**Figure 4:**
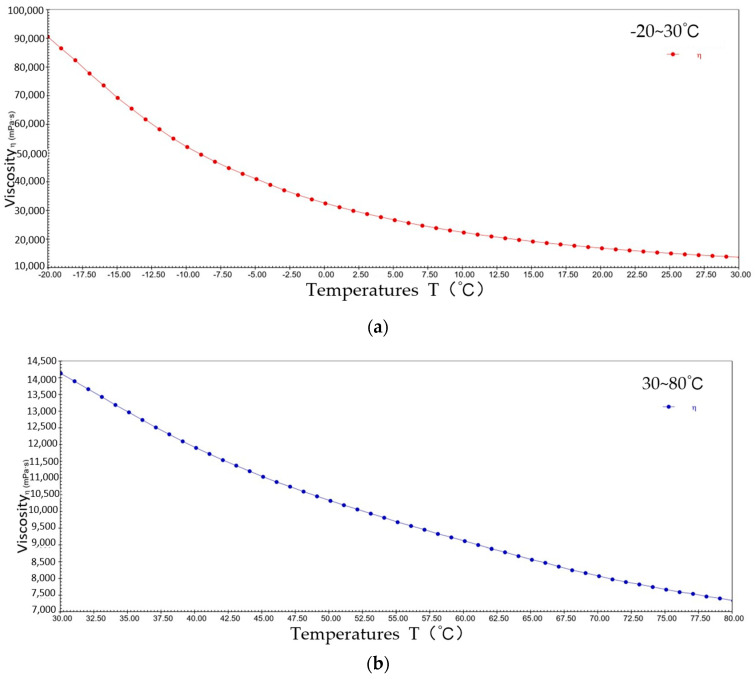
The viscosity–temperature curve for sample 1. (**a**) −20~30 °C; (**b**) 30~80 °C.

Figure 4 shows the pattern of change in viscosity with a temperature rise for sample 1 at a constant shear rate. The viscosity decreased from 9.1 × 10^4^ mPa·s to 1.4 × 10^4^ mPa·s at −20~30 °C; the viscosity decreased from 1.4 × 10^4^ mPa·s to 7.3 × 10^3^ mPa·s at 30~80 °C. It can be seen that during the warming process, sample 1 presents typical viscosity–temperature characteristics, i.e., the viscosity decreases with increasing temperature. This is due to elevated temperatures increasing the thermal energy of the lubricant molecules, giving them a greater average kinetic energy, resulting in weaker interactions between the molecules of sample 1 and the molecules sliding relatively freely in their motion, thus reducing viscosity.

#### 2.1.2. Effects of RFM3000 on the Rheological Characteristics of the Gel-State Mobil SHC 461WT Grease

**Figure 5:**
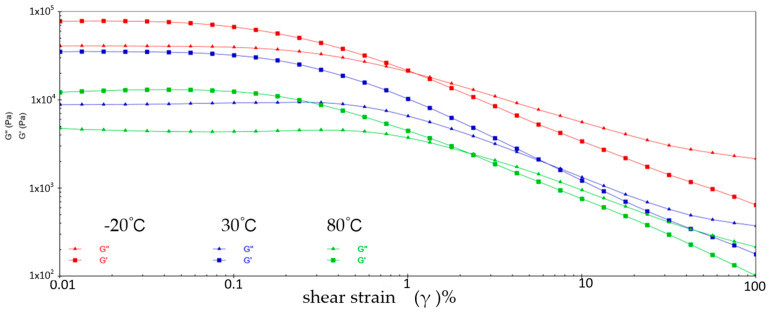
The viscoelastic modulus–shear strain curve for sample 2.

Figure 5 shows the variation patterns of viscoelastic modulus versus shear strain for sample 2 at three different temperatures. At −20 °C, the strain value at the flow point of sample 2 is 1.12% and G′ = G″ = 1.9743 × 10^4^ Pa; at 30 °C, the strain value at the flow point of sample 2 is 6.429% and G′ = G″ = 1.863 × 10^3^ Pa; at 80 °C, the strain value at the flow point of sample 2 is 2.213% and G′ = G″ = 2.509 × 10^3^ Pa. It can be seen that after the addition of RFM3000, the flow point strain values of sample 2 are lower than those of sample 1 at both −20 °C and 80 °C, but slightly higher at 30 °C. This is because the addition of metallic molybdenum additives improves the high- and low-temperature performance of the grease. It is capable of preventing sample 2 from becoming overly viscous at low temperatures as well as preventing sample 2 from becoming excessively diluted at high temperatures, thereby reducing the deformation value of the flow point at −20 °C and 80 °C. The property of molybdenum metal to enhance high- and low-temperature performance is weakened in the room temperature environment at 30 °C, and, therefore, the flow point strain value of sample 2 is elevated.

**Figure 6:**
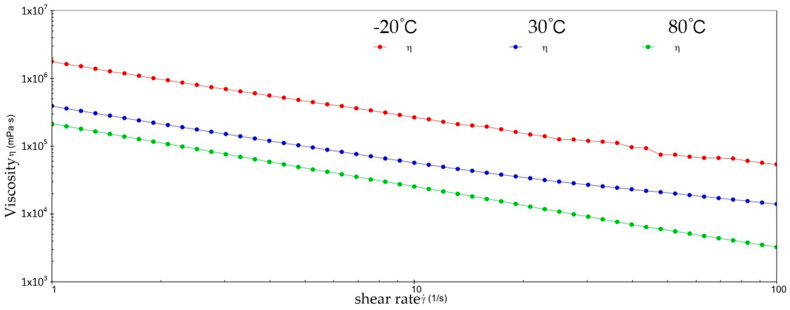
The viscosity–shear rate curve for sample 2.

**Figure 7:**
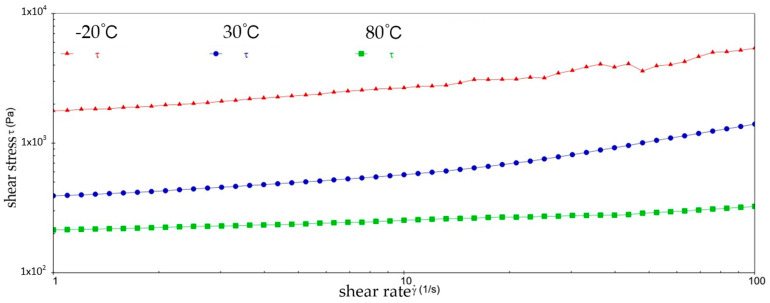
The shear stress–shear rate curve for sample 2.

Figure 6 and Figure 7 show the variation patterns of viscosity and shear stress with shear rate for sample 2. At −20 °C, the viscosity of sample 2 decreased from 1.78 × 10^6^ mPa·s to 5.4 × 10^4^ mPa·s and the shear stress increased from 1.7759 × 10^3^ Pa to 5.3962 × 10^3^ Pa; at 30 °C, the viscosity of sample 2 decreased from 3.92 × 10^5^ mPa·s to 1.4 × 10^4^ mPa·s, and the shear stress increased from 3.9211 × 10^2^ Pa to 1.4016 × 10^3^ Pa; at 80 °C, the viscosity of sample 2 decreased from 2.1 × 10^5^ mPa·s to 3.25 × 10^3^ mPa·s, and the shear stress increased from 2.1385 × 10^2^ Pa to 3.2492 × 10^2^ Pa. The decreasing trend of viscosity with the shear rate for sample 2 slows down significantly at 30 °C but accelerates at −20 °C ambient temperature. This is due to the addition of a metallic molybdenum additive at low temperatures, which improves the low-temperature performance of the grease, resulting in a lower viscosity for sample 2. When the shear rate increases, the viscosity of the grease decreases more significantly. In addition, the increase in shear stress for sample 2 was less than that for sample 1 at −20 °C and 80 °C, while the opposite was true at 30 °C. This proves that the gel-state grease with RFM3000 above has excellent flow characteristics in high- and low-temperature environments.

**Figure 8:**
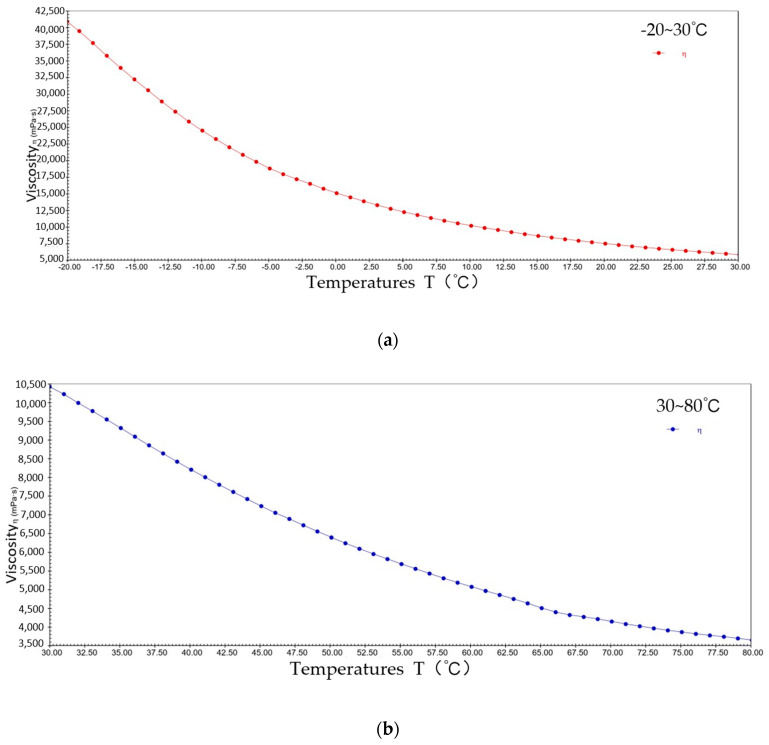
The viscosity–temperature curve for sample 2. (**a**) −20~30 °C; (**b**) 30~80 °C.

Figure 8 shows the typical viscosity–temperature characteristics of sample 2. At −20 °C to 30 °C, the viscosity of sample 2 decreased from 1.2 × 10^4^ mPa·s to 2.7 × 10^3^ mPa·s; at 30 °C to 80 °C, the viscosity of sample 2 decreased from 2.1 × 10^4^ mPa·s to 4.8 × 10^3^ mPa·s. The initial and final viscosities of both experiments were less than those of sample 1, again indicating that sample 2 has better flow characteristics.

#### 2.1.3. Effect of SK3115 on the Rheological Characteristics of the Gel-State Mobil SHC 461WT Grease

**Figure 9:**
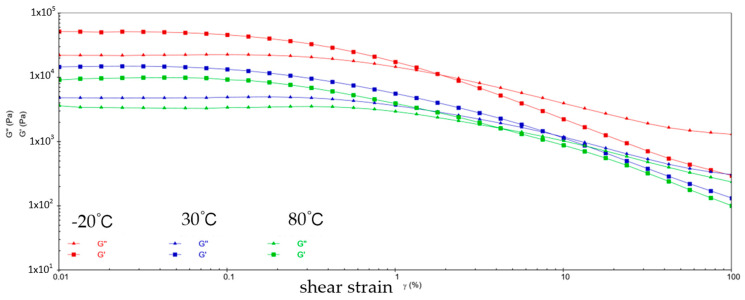
The viscoelastic modulus–shear strain curve for sample 3.

Figure 9 shows the variation patterns of viscoelastic modulus versus shear strain for sample 3 at three different temperatures. At −20 °C, the flow point strain value of sample 3 is 1.298% and G′ = G″ = 2.0832 × 10^4^ Pa; at 30 °C, the flow point strain value of sample 3 is 2.464% and G′ = G″ = 4.022 × 10^3^ Pa; at 80 °C, the flow point strain value of sample 3 is 0.979%, G′ = G″ = 5.028 × 10^3^ Pa. It can be seen that the flow point strain values of sample 3 at three different temperatures are less than that of sample 1. This is due to the fact that the boronized rare earth nanoparticles in SK3115 can effectively reduce the viscosity of the grease. The smaller size of the nanoparticles allows them to penetrate between the molecular chains of the grease and improve the flow of the grease, thus reducing the strain value of the grease at the point of flow.

**Figure 10:**
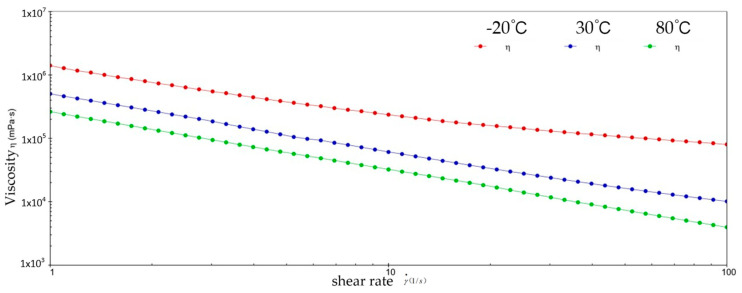
The viscosity–shear rate curve for sample 3.

**Figure 11:**
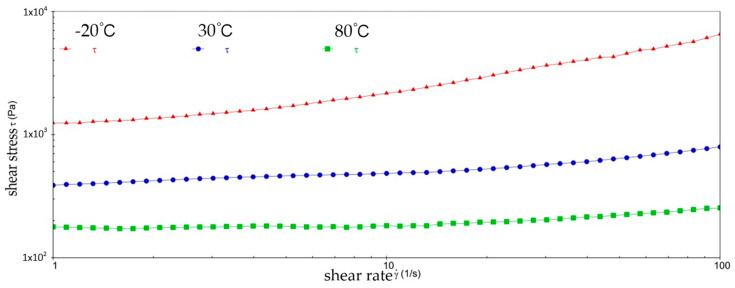
The shear stress–shear rate curve for sample 3.

Figure 10 and Figure 11 show the variation patterns of viscosity and shear stress with shear rate for sample 3. At −20 °C, the viscosity of sample 3 decreased from 1.24 × 10^6^ mPa·s to 6.5 × 10^4^ mPa·s, and the shear stress increased from 1.2413 × 10^3^Pa to 6.5083 × 10^3^ Pa; at 30 °C, the viscosity of sample 3 decreased from 3.87 × 10^5^ mPa·s to 7.9 × 10^3^ mPa·s, and the shear stress increased from 3.8731 × 10^2^ Pa to 7.9394 × 10^2^ Pa; at 80 °C, the viscosity of sample 3 decreased from 1.78 × 10^5^ mPa·s to 2.54 × 10^3^ mPa·s, and the shear stress increased from 1.7813 × 10^2^ Pa to 2.5376 × 10^2^ Pa. It can be seen that more pronounced fluctuations in the course of the viscosity and shear stress of sample 3 were observed at −20 °C and 80 °C. On the one hand, this is due to the fact that nanoborated rare earths have a certain crystal structure and shape. Its effect is to form a three-dimensional network structure in sample 3, which improves the load-bearing capacity and extreme pressure performance of sample 3. This structure may be stable at a normal temperature. But at low temperatures, the crystal structure of the boronated rare earth nanoparticles is altered, leading to the restriction of the mobility of sample 3. In addition, the change in shear rate leads to the structural rearrangement of sample 3, and the presence of borated rare earth nanoparticles triggers an inhomogeneous or nonlinear response in the process, resulting in the fluctuation phenomenon. On the other hand, partial melting or aggregation of nanoborated rare earths may occur at high temperatures, which will cause changes in the internal structure of sample 3. The aggregation phenomenon causes the nanoparticles or crystal structure in sample 3 to rearrange, which results in fluctuations in the viscosity and shear stress of sample 3. This suggests that the unstable viscosity change of sample 3 at different shear rates may lead to unstable friction and wear properties of the mechanical components, thus increasing the risk of friction, wear, and failure of the mechanical components. Especially at −20 °C and 80 °C, its lubricant performance may not be reliable enough to effectively provide the required lubrication protection.

**Figure 12:**
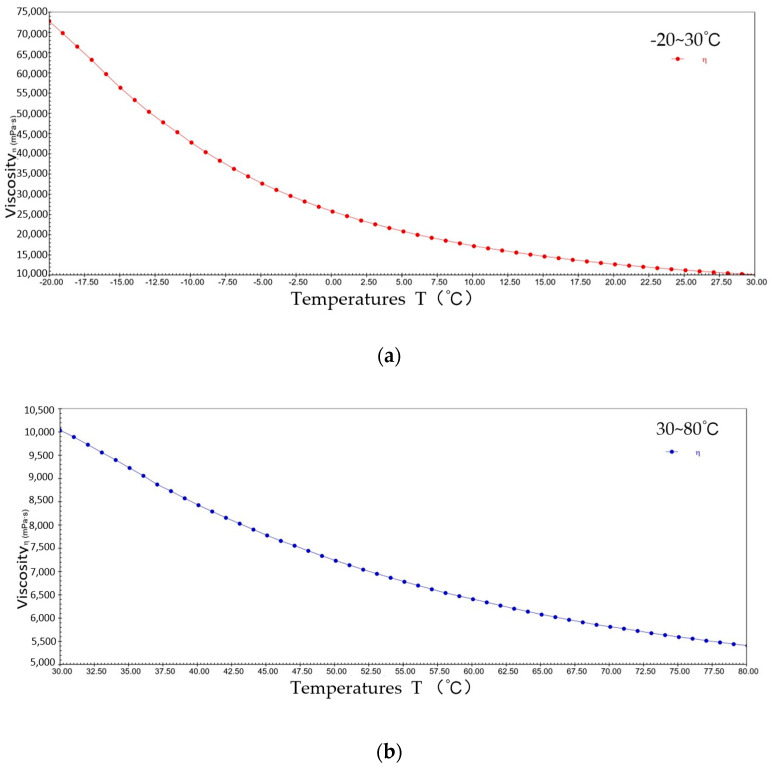
The viscosity–temperature curve for sample 3. (**a**) −20~30 °C; (**b**) 30~80 °C.

Figure 12 shows the viscosity–temperature profile of sample 3. At −20 °C to 30 °C, the viscosity decreased from 4.1 × 10^4^ mPa·s to 5.8 × 10^3^ mPa·s; at 30 °C to 80 °C, the viscosity decreased from 1.04 × 10^4^ mPa·s to 3.6 × 10^3^ mPa·s. It can be seen that in the temperature interval of −20 °C to 30 °C, the viscosity drop of sample 3 is relatively overly large and its adhesion may be affected. This will result in sample 3 not adhering effectively to the surface of the mechanical components, thereby increasing the risk of frictional wear, failure, and grease leakage from the wind turbine. The viscosity–temperature curve does not decrease smoothly between 30 °C and 80 °C, which also indicates that sample 3 is subject to lubrication instability as the ambient temperature rises to high temperatures.

#### 2.1.4. Effect of PV611 on the Rheological Characteristics of the Gel-State Mobil SHC 461WT Grease

**Figure 13:**
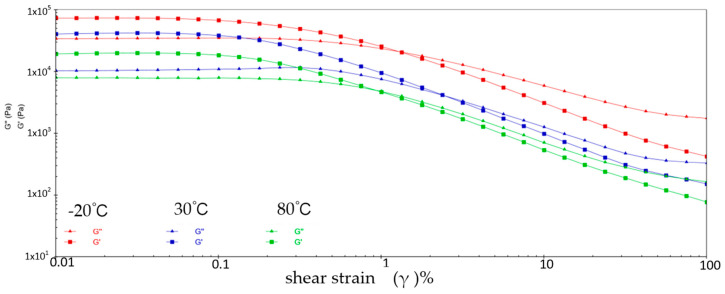
The viscoelastic modulus–shear strain curve for sample 4.

Figure 13 shows the correspondence between viscoelastic modulus and shear strain for sample 4. At −20 °C, the flow point strain value of sample 4 was 1.841% and G′ = G″ = 1.0987 × 10^4^ Pa; at 30 °C, the flow point strain value of sample 4 was 8.56% and G′ = G″ = 1.298 × 10^3^ Pa; at 80 °C, the flow point strain value of sample 4 is 4.198% and G′ = G″ = 1.615 × 10^3^ Pa. It can be seen that only at −20 °C, the flow point strain value of sample 2 decreases with respect to sample 1, while at the other two temperature conditions, the flow point strain values are reversed. Especially at 30 °C, the flow point strain value increases dramatically. This is due to the fact that the additive PV611 contains fatty acid compounds, molecules of which can interact with the base oil and other types of pre-existing additives in sample 4, thus affecting the performance of sample 4. At −20 °C, the additive PV611 was able to increase the flowability of sample 4, resulting in a decrease in the flow point strain value of sample 4 at low temperatures. This is due to the ability of the fatty acid compounds to improve the low-temperature fluidity of sample 4, thereby reducing the viscosity and flow point strain values of sample 4. However, at room temperature of 30 °C or a high temperature of 80 °C, it may react with the base oil and additives in sample 4 to form larger molecular polymers or polymers that increase the viscosity of the grease, which leads to an increase in the value of the flow point strain. This phenomenon may result in uneven distribution of sample 4 on the surface of the mechanical component, or insufficient lubrication in certain areas to provide long-lasting lubrication, resulting in localized friction and wear of the mechanical component during operation and increasing the risk of equipment failure.

**Figure 14:**
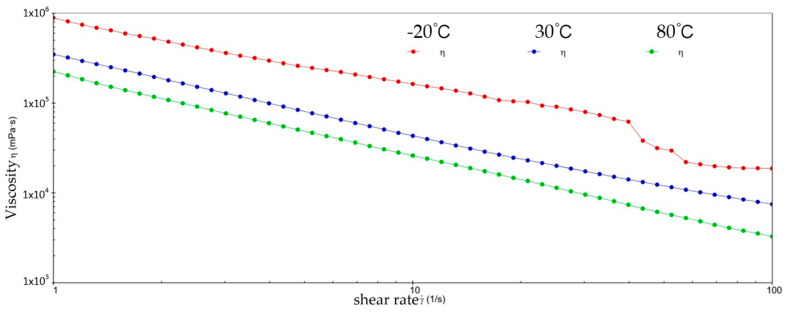
The viscosity–shear rate curve for sample 4.

**Figure 15:**
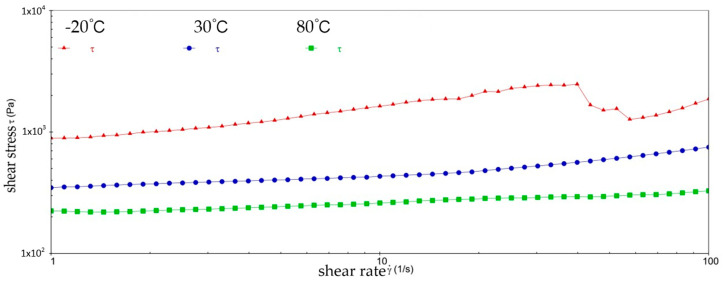
The shear stress–shear rate curve for sample 4.

Figure 14 and Figure 15 show the variation patterns of viscosity and shear stress with shear rate for sample 4. At −20 °C, the viscosity of sample 4 decreased from 8.89 × 10^5^ mPa·s to 1.87 × 10^4^ mPa·s, and the shear stress increased from 8.8844 × 10^2^ Pa to 1.8678 × 10^3^ Pa; at 30 °C, the viscosity of sample 4 decreased from 3.48 × 10^5^ mPa·s to 7.5 × 10^3^ mPa·s, and the shear stress increased from 3.4794 × 10^2^ Pa to 7.4938 × 10^2^ Pa; at 80 °C, the viscosity of sample 4 decreased from 2.24 × 10^5^ mPa·s to 3.28 × 10^3^ mPa·s, and the shear stress increased from 2.2419 × 10^2^ Pa to 3.2787 × 10^2^ Pa. At −20 °C and with the shear rate around 41 s^−1^, sample 4 showed a significant drop in viscosity and shear stress. This is caused by the combination of temperature and additives. At −20 °C, the experimental initial viscosity of sample 4 decreased substantially. Meanwhile, as the shear rate increased, the fatty acid analogs in PV611 interfered with the molecular arrangement in sample 4. At low temperatures, these interactions may be enhanced, leading to an easier flow of molecules in sample 4 and exacerbating shear dilution. Thus, there is a sudden drop in viscosity and shear stress. Moreover, the overall viscosity of sample 4 showed a significant decrease under three different temperatures. It can be seen that although PV611 improves the fluidity performance of the grease, the viscoelasticity is not guaranteed as it should be. This means that the thickness of its lubrication film will be reduced, and it will not be able to effectively isolate the contact between metal surfaces. The low viscosity may also lose the stability of the lubricating film during the operation of the mechanical equipment and may be easily extruded or lost. This will result in the lubrication effect not being sustained, triggering insufficient lubrication and damage to parts during high- and low-temperature operation of the equipment.

**Figure 16:**
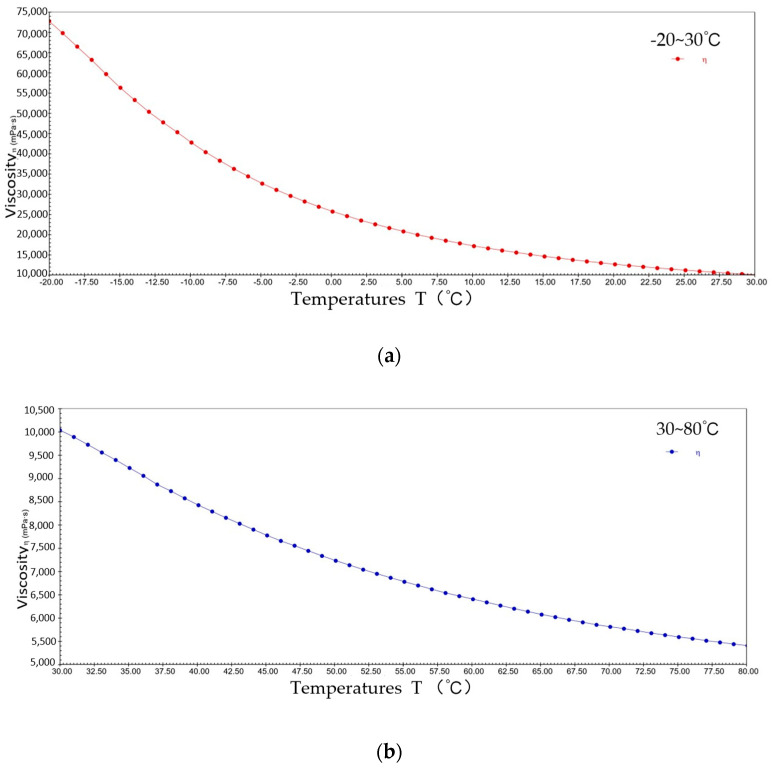
The viscosity–temperature curve for sample 4. (**a**) −20~30 °C; (**b**) 30~80 °C.

Figure 16 shows the viscosity–temperature profile of sample 4. At −20 °C to 30 °C, the viscosity decreased from 7.2 × 10^4^ mPa·s to 1 × 10^4^ mPa·s; at 30~80 °C, the viscosity decreased from 1 × 10^4^ mPa·s to 5.4 × 10^3^ mPa·s. It can be seen that the viscosity of sample 4 decreases by about 86% in the interval from −20 °C to 30 °C, which fully proves that the grease has poor adhesion after the addition of PV611 and is unable to maintain a stable lubrication film during the warming process.

### 2.2. Comparative Experimental Rheological Analysis of the Gel-State of Mobil SHC 461WT Grease with Different Additives

#### 2.2.1. Analysis of Three Different Additives on the Viscoelastic Characteristics of the Gel-State of Mobil SHC 461WT Grease

**Figure 17:**
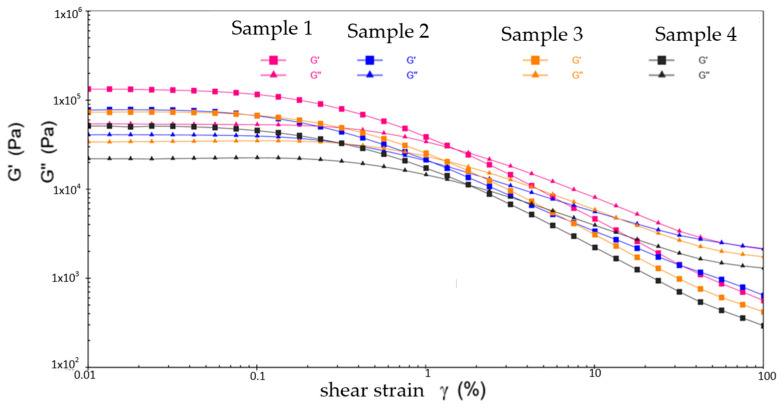
The viscoelastic modulus–shear strain curves for four different samples at −20 °C.

**Figure 18:**
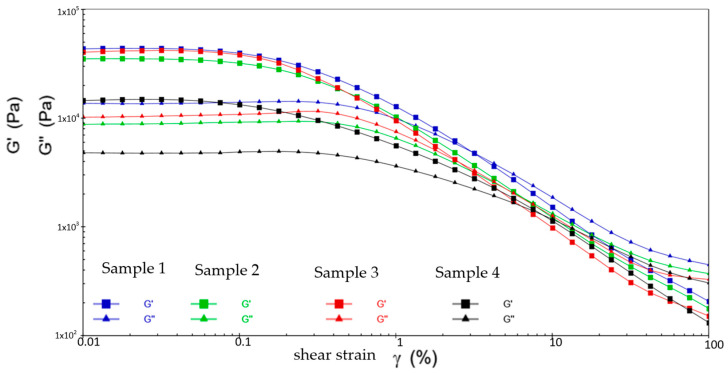
The viscoelastic modulus–shear strain curves for four different samples at 30 °C.

**Figure 19:**
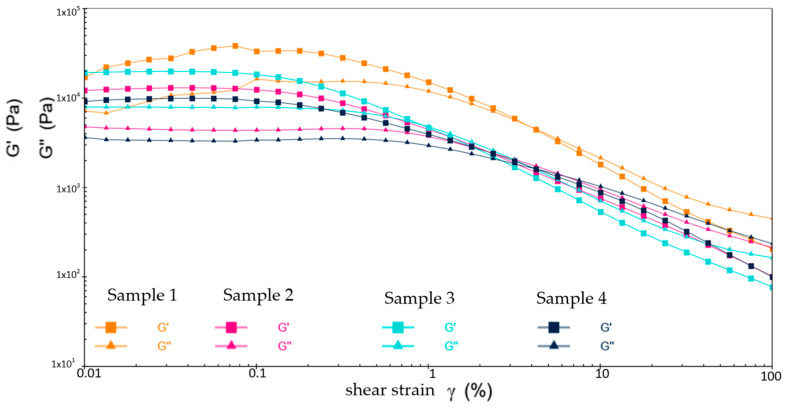
The viscoelastic modulus–shear strain curves for four different samples at 80 °C.

Figure 17, Figure 18 and Figure 19 show the viscoelastic modulus versus shear strain for four different samples at different temperatures. From Figure 17, at −20 °C, samples 1, 2, 3, and 4 show only slight differences in flow point strain values of 1.538%, 1.12%, 1.298%, and 1.841%, respectively. Compared to SK3115 and PV611, the RFM3000 additive significantly improves the viscoelasticity of sample 2 at low temperatures. It effectively overcomes the difficulty of the high viscosity of sample 2 caused by low temperature, thus inducing the formation of an effective lubricating film on sample 2. This allows mechanical parts to be lubricated more quickly, reducing friction and wear at startup, thus improving the startup performance of the equipment. Meanwhile, sample 2 achieves uniform lubrication in extreme environments, smoother equipment operation, and reduced noise and vibration, contributing to stable equipment operation and efficiency.

From Figure 18, at 30 °C, the flow point strain values for samples 1, 2, 3, and 4 are 3.118%, 6.429%, 2.464%, and 8.56%, respectively. Sample 3 shows the best viscoelastic properties. However, from the values of G′ and G″, the PV611 additive significantly reduced G′ and G″ of sample 4. This causes the structure of sample 4 to become unstable and unable to effectively withstand shear forces, which in turn leads to a loss of mass of sample 4 and reduces its adhesion and durability on the surface of the mechanical component. It may also cause oil molecules to be released from sample 4, reducing the effective lubricating component, which may result in increased oil consumption and the need for the equipment to add or replace grease more frequently.

From Figure 19, at 80 °C, the flow point strain values for samples 1, 2, 3, and 4 are 3.826%, 2.213%, 0.979%, and 4.198%, respectively. The viscoelastic modulus of sample 1 shows significant fluctuations, indicating a lack of thermal stability at high temperatures. This may initiate phenomena such as chemical reactions, decomposition, or aging, which may alter the lubricating characteristics of sample 1 and may even render it ineffective for lubrication. Meanwhile, the other three samples have relatively stable curves at high temperatures. In addition, RFM3000 moderately reduces the flow point strain value of sample 2, while SK3115 causes sample 3 to almost completely lose its viscoelastic characteristics in high-temperature environments, making it exceptionally easy to flow. This can lead to increased wear rates on the equipment during operation due to frictional direct contact at high-velocity contact surfaces.

Above all, the viscoelastic modulus of the four different samples showed a decreasing trend with increasing temperature, which is in line with the shear dilution principle of grease. At −20 °C and 80 °C, RFM3000 significantly reduced the flow point strain value of sample 2 with the best viscoelastic performance enhancement; at 30 °C, the best performance in terms of rheological characteristics was achieved by SK3115, but it had sufficient conditions to enable the equipment to fail and therefore was not sufficient to reduce the energy consumption of the wind turbine during operation. The PV611 additive increased the flow point strain value of sample 4 at all three temperatures, making sample 4 excessively viscous and simultaneously increasing the energy consumption of the equipment throughout its life cycle.

#### 2.2.2. Analysis of Three Different Additives on the Flow Characteristics of the Gel-State Mobil SHC 461WT Grease

**Figure 20:**
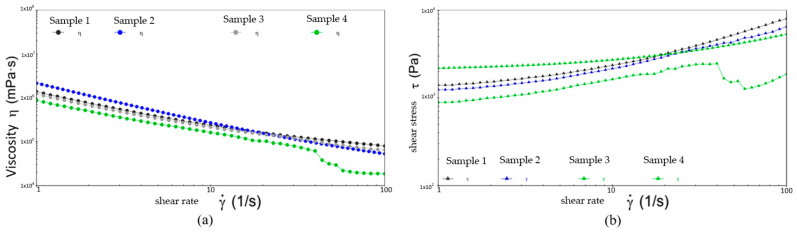
The viscosity–shear rate and shear stress–shear rate curves for four different samples at −20 °C. (**a**) Viscosity-shear rate; (**b**) Shear stress-shear rate.

**Figure 21:**
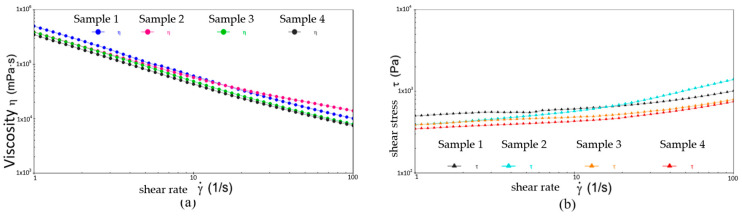
The viscosity-shear rate and shear stress-shear rate curves for four different samples at 30 °C. (**a**) Viscosity-shear rate; (**b**) Shear stress-shear rate.

**Figure 22:**
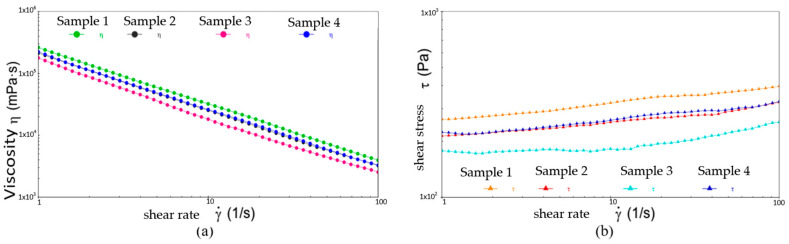
The viscosity-shear rate and shear stress-shear rate curves for four different samples at 80 °C. (**a**) Viscosity-shear rate; (**b**) Shear stress-shear rate.

**Figure 23:**
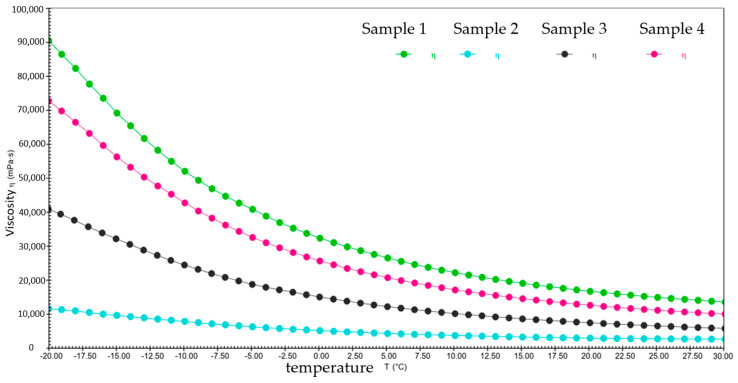
The viscosity–temperature curves for four different samples from −20 °C to 30 °C.

Figure 20, Figure 21 and Figure 22 show the comparative analysis of viscosity, shear stress, and shear rate curves for four different samples at different temperatures. From Figure 22, at −20 °C, the viscosities of samples 1, 2, and 3 show a uniform decreasing trend with increasing shear rate, while the shear stresses also show a uniform increasing trend, indicating excellent low-temperature fluidity. On the contrary, sample 4 shows inferior performance at higher shear rates, indicating susceptibility to yield damage at low temperatures and high shear rates. Figure 23 shows that either sample exhibits excellent flow properties at 30 °C. In addition, the values of initial viscosity, shear stress, and shear rate up to 100 s^−1^ differ slightly among the four different samples, indicating that they have similar flow stability at room temperature.

Figure 22 shows the defects of sample 3 in the high-temperature environment. At the shear rate of 0 s^−1^, its viscosity is the smallest among the four samples. As the shear rate rises to 100 s^−1^, the viscosity is still minimal and the shear stress to be applied is similarly small and less stable. This is ample evidence of the presence of flow anomalies in high-temperature environments. In addition, although the viscosities of sample 2 and sample 3 are slightly lower than that of sample 1, the shear stresses that need to be applied are drastically less than those of sample 1. This suggests that the application of samples 2 and 3 can effectively reduce energy consumption and increase the efficiency of the wind turbine when the viscosities are similar. As a result, they extend the lifespan of the equipment and reduce the frequency of repairs and replacement parts during the long-term operation of the equipment.

Following the above, the grease of sample 2 performed the best at three different temperatures. At 80 °C, sample 3 shows an abnormal flow behavior, resulting in insufficient stability of the resulting lubricant film, which loses the desired effect of the grease. At −20 °C, sample 4 shows shear yielding, which makes it structurally unstable and less mobile at low temperatures.

#### 2.2.3. Analysis of Three Different Additives on the Viscosity–Temperature Characteristics of the Gel-State Mobil SHC 461WT Grease

Figure 23 and Table 2 show the viscosity–temperature curves and comparison of viscosity changes for four different samples from −20 °C to 30 °C, respectively. Under the constant shear rate and low-temperature conditions, the viscosity–temperature curves of the four different samples decreased smoothly, and all of them showed good temperature stability. Combined with the viscosity change data in Table 2, sample 2 has a lower starting and ending viscosity compared to the other three samples. This shows that sample 2 is effective in reducing adhesion, friction, and energy loss at low temperatures, thus improving the efficiency of the wind turbine.

**Figure 24:**
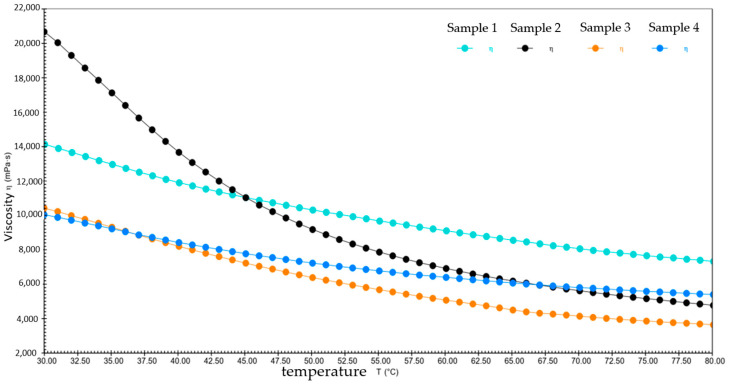
The viscosity–temperature curves for four different samples from 30 °C to 80 °C.

Figure 24 and Table 3 show the comparison of viscosity–temperature curves and viscosity changes of four different samples at 30 °C to 80 °C, respectively. The relatively high initial viscosity of sample 2 during the warming process from 30 °C to 80 °C ensures its excellent lubrication under the room temperature environment. As the temperature rises, its viscosity decreases significantly and it is able to maintain relatively low viscosity at high temperatures, which helps maintain lubrication in high-temperature environments, even when temperatures change dramatically. The remaining three samples have lower and slower decreasing initial viscosities, which do not change much with low-temperature conditions. This indicates that they show weak flow ability at high temperatures and insufficient lubrication, causing problems such as overheating, welding, sticking, and wearing of equipment, leading to increased equipment repair and replacement costs.

Overall, sample 2 exhibits excellent flow properties at two different temperature rise environments, while the other three samples have relatively inferior flow characteristics at high and low temperatures. Therefore, it can be assumed that sample 2 provides a reliable lubrication guarantee for industrial applications in various temperature environments.

#### 2.2.4. Rheological Modeling

The rheological characterization of non-Newtonian fluids is a key research direction in the rheological study of gel-state greases. To quantitatively describe the flow law of gel-state grease, the Herschel–Bulkley (H–B) model is used in this paper to curve-fit the parameters of four different samples. The fitting equation is as follows:

$$\tau =\tau_{0}+k(\dot{\gamma }{)}^{n}$$

In the formula, $\tau_{0}$ is the yield stress of the gel-state grease, k is the flow behavior index, n is the shear thinning index, and $\dot{\gamma }$ is the shear rate of the gel-state grease. Based on the obtained data on the rheological characteristics of the gel-state grease, the trends of the apparent viscosity with shear rate at different temperatures for four different samples can be effectively investigated. By using the rheological data obtained in continuous shear mode, it can be observed that the measured data are in reasonable agreement with the obtained curves. Therefore, the rheological equations are fitted using the experimental data and the parameter values of the H–B model are calculated to characterize the changing patterns of the shear stress curves of the four different samples. The rheological fitting equations for four different samples at different temperatures are shown in Table 4.

**Figure 25:**
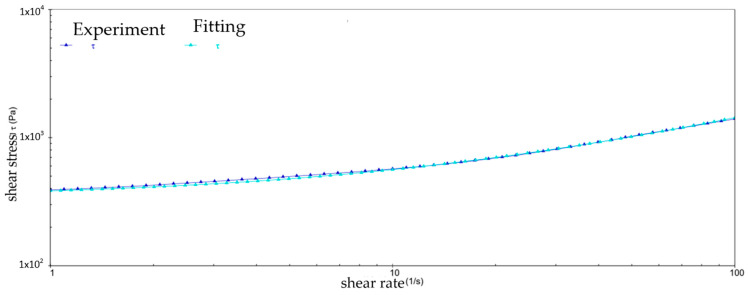
Comparison of the fitting equations and experimental data for sample 2 at 30 °C.

According to the fitting results, there is a better match between the rheological model and the experimental data, as shown in Figure 25. This shows that the rheological parameters are capable of describing the rheological behavior of the grease by means of rheological modeling, which in turn allows the derivation of the eigenequations for four different samples at different temperatures.

## 3. Conclusions

### 3.1. Conclusions

In this study, the rheological characteristics of four different samples of gel-state greases are analyzed, and their viscoelastic, flow, and viscosity–temperature characteristics are discussed in depth. Comprehensive analysis of the experimental data and the H–B model leads to the following conclusions:

(1) The best additive to improve the overall lubricating performance of the gel-state grease is the RFM3000 additive. In rheological experiments, the additive maintains the molecules of sample 2 in a stable state, which can effectively adapt to various temperature environments and maintain the stability of the internal structure. As the temperature rises, it ensures that the sample maintains excellent fluidity. This highlights that additive RFM3000 further reduces friction and drag in existing gel-state wind turbine greases, reducing the amount of energy required inside wind turbines. This will help to reduce power loss in the power generation process and reduce the energy consumption of the whole wind turbine, thus further reducing carbon emissions.
(2) Sample 3 has lower values of flow point strain only at 30 °C than at other temperatures, while the rheological characteristics are less promising at other temperatures than the other samples. Although the rheological characteristics of sample 4 are slightly better than those of sample 1, there is still a large gap compared to sample 2. Above all, both samples 3 and 4 are not suitable as the gel-state grease for 1.5 MW large horizontal axis wind turbines.
(3) The H–B model can effectively reveal the sensitivity of gel-state grease to temperature changes. The intrinsic equations of the gel-state grease obtained by the H–B model can effectively predict the rheological characteristics of the gel-state grease at different temperatures, which provides a reasonable reference for the rheological characteristics of the gel grease under different conditions.

### 3.2. Future Perspectives

(1) Regarding the problem of how to reduce the carbon emissions of 1.5 MW large-scale horizontal axis wind turbines, this paper only investigates and optimizes the performance of the gel-state grease. The wind turbine in the actual work operation process, the structure of the components of the force, material, load, and other factors will also affect the efficiency of the wind turbine and failure rate. Therefore, the follow-up work needs to take full account of the impact of these factors and conduct a more comprehensive analysis.
(2) Regarding the performance improvement of additives for the gel-state grease, the RFM3000 additive, which has the best performance improvement effect in this paper, also has some problems in the experiment. In future research, the selection and application of other additives can be explored to minimize their adverse environmental impacts and further improve the eco-friendliness of gel-state greases. Meanwhile, it is necessary to introduce equipment such as a four-ball friction and wear tester and SRV tester to test the tribological wear of the optimized gel-state grease, which is the direction of our further research.

## 4. Materials and Methods

### 4.1. The Rheological Performance Test Program for the Gel-State Mobil SHC 461WT Grease

#### Experimental Equipment

**Figure 26:**
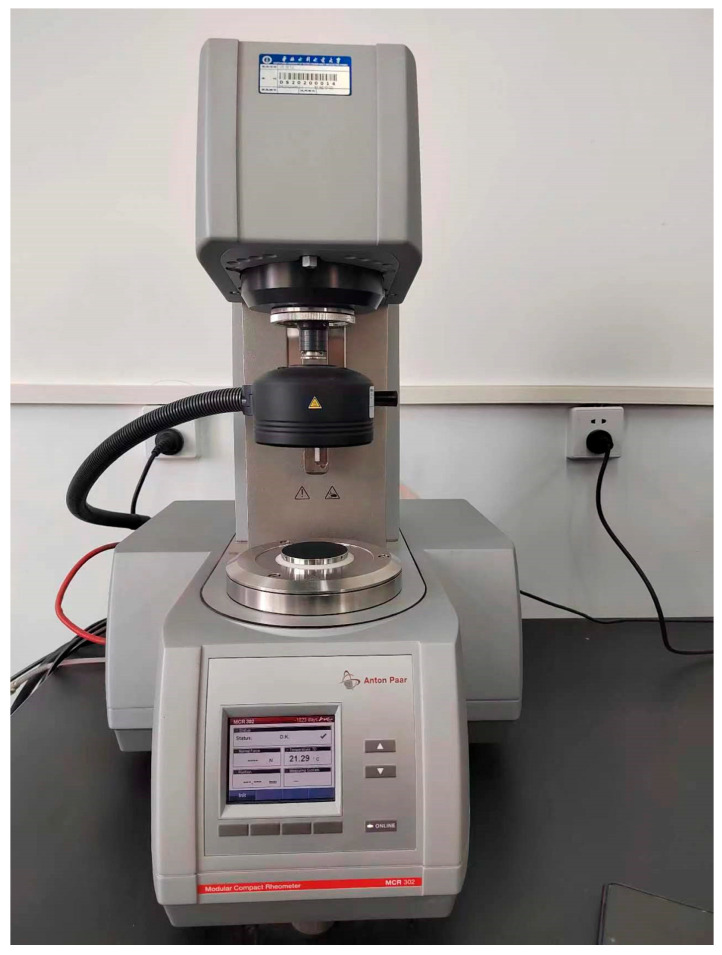
The Anton Paar MCR302 rotational rheometer (Anton Paar, Ashland, VA, USA).

The Anton Paar MCR302 rotational rheometer can be used to measure a wide range of parameters such as shear stress, shear rate, viscosity, and rheological profile of gel-state materials such as liquids and soft solids (The Anton Paar MCR302 Rotational Rheometer manufactured by Anton Paar Co. (Shanghai, China)). Its rotational speed ranges from 10^−6^~200 r/min, measuring torque from 10 μ N·m to 0.2 μ N·m, and the normal force ranges from 0.001 N to 50 N to evaluate the rheological characteristics of different materials [34]. Its built-in RheoCompass application analyzes, plots, and exports experimental data in real-time for a more comprehensive assessment of the rheological characteristics of materials and provides a wide range of rheological models and terminology for working with complex rheological data and making rheological property predictions. This device is shown in Figure 26.

### 4.2. Rheological Experimental Design and Parameterization

**Figure 27:**
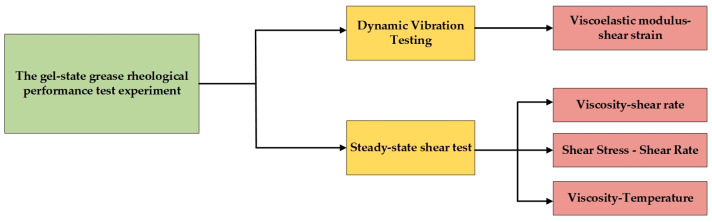
The test protocol for the rheological characteristics of the gel-state Mobil SHC 461WT grease.

Due to the 1.5 MW large-scale horizontal axis, wind turbines usually work in harsh, wide temperature ranges and other environments. Temperature is one of the most important factors affecting the flow point strain value of wind-power gel-state greases [36]. As the temperature changes, the flow characteristics of gel-state greases change significantly [37]. At higher temperatures, the fluidity of the gel grease increases and the flow point strain value decreases, which may lead to premature flow of the gel-state grease, affecting the lubrication effect and service lifespan of the wind turbine. In contrast, at low temperatures, the fluidity of the gel-state grease is weakened, and the flow point strain value is increased, which may lead to excessive viscosity and affect the start-up and normal operation of the wind turbine. According to the survey, it is known that the standard operating temperature range of wind turbines is usually between −20 °C and 80 °C, and the operating temperature of bearings during operation does not exceed 80 °C [38]. Therefore, the temperature measurement points chosen in this paper are −20 °C, 30 °C, and 80 °C. In the dynamic oscillation test, the control angular velocity was kept constant at 10 rad/s and the shear strain ranged from 0.1% to 100%. In the steady state shear tests, the viscosity–temperature experiments control the shear rate to be 50 s^−1^, and the viscosity–shear experiments, for example, control the shear rate in the range of 0.01 s^−1^ to 100 s^−1^. The specific experimental program of this research is shown in Figure 27.

### 4.3. Selection of Grease and Additives

**Figure 28:**
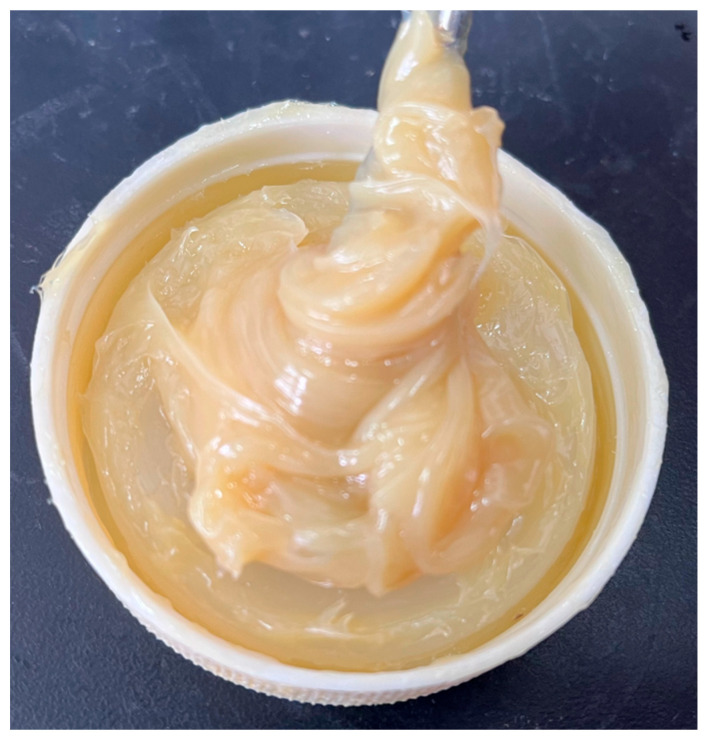
The gel state of Mobil SHC 461WT grease.

Due to the harsh working environment of wind turbines, the grease used is required to have excellent water resistance, high-temperature resistance, oxidation resistance, corrosion resistance, and other characteristics. Therefore, when selecting grease, it is necessary to consider a variety of factors, such as the working environment, operating conditions, bearing materials, and utilization requirements. In this paper, according to the wind turbine grease type and parameters, the gel-state Mobil SHC 461WT grease is selected, as shown in Figure 28. The gel-state of Mobil SHC 461WT is the most common grease on the market and is widely used in the field of wind turbine lubrication. It adopts advanced polyurethane base oil and additive technology, with excellent oxidation stability, heat resistance, water resistance, and high bearing capacity, which can meet the operational requirements of wind turbine spindle bearings (The gel state Mobil SHC 461WT grease is manufactured by China, Hubei Litu Industrial Co., Jingmen, China). The three different additives selected are RFM3000 (the main component of RFM3000 is molybdenum dialkyl dithiocarbamate (MoDTC), which has excellent chemical stability, high decomposition temperature, and high viscosity; especially, the MoDTC’s anti-wear performance is more outstanding under high load or high-temperature conditions), SK3115 (SK3115 nano-boronated rare-earth friction modifier is prepared by a chemical reaction method, which is characterized by low cost and light color; compared with traditional sulfur and phosphorus friction reducers, it is less toxic and has better high-temperature resistance and corrosion resistance), and PV611 (PV611 friction modifier is a fatty based friction reducer produced by Lubrizol; it is rated for a wide range of certifications and is used in a wide variety of applications).

For the convenience of experimental analysis, sample 1 represents the gel state of Mobil SHC 461WT grease, sample 2 represents the gel state of Mobil SHC 461WT grease with the addition of RFM3000, sample 3 represents the gel state of Mobil SHC 461WT grease with the addition of SK3115, and sample 4 represents the gel state of Mobil SHC 461WT grease with the addition of PV611. To make this study more informative, the additive content in samples 2, 3 and 4 is 0.1% [39].

## Figures and Tables

**Table 1:** The progress made by researchers on additives in lubricants.

Reference Number	Author(s), Year	Major Findings
[22]	Mousavi et al., 2020	The addition of ZnO nanoparticles to diesel fuel was found to significantly improve its tribological characteristics
[23]	Mousavi et al., 2019	The friction and wear tests revealed that MoS_2_ had no significant effect on the anti-wear performance of diesel fuel.
[24]	Xiong et al., 2023	It was demonstrated that fluorinated graphite and fluorinated graphene had a significant effect on the taper inlet, stencil oil separation, and evaporation loss of polyurea grease.
[25]	Liang et al., 2023	It was found that calcium carbonate nanoparticles can improve the anti-wear and friction reduction characteristics of grease.
[26]	Xu et al., 2023	Various gel-state greases were prepared, and their characteristics were improved.
[27]	Dai et al., 2024	Improved application of ester lubricants in weak-gel drilling fluids.
[28]	Jopen et al., 2023	Comparison of polyester organogel formation in three different base oils.
[29]	Schwack et al., 2020	It was concluded that grease lubricants with low base oil viscosity and high osmolality have higher anti-wear characteristics.
[30]	Ng et al., 2020	It was found that graphene nanoparticles can significantly reduce the friction and wear of bearings.
[31]	Saidi et al., 2021	It was found that MoS_2_ nano-additives have high antiwear and friction reduction capabilities.
[32]	Wu et al., 2022	The additives of hexagonal boron nitride and calcium carbonate nanoparticles as base oils were prepared.
[33]	Li et al., 2023	A gel grease for wind turbine generator bearings was prepared.
[34]	Rosenkranz et al., 2021	Four different gel greases are produced on the basis of mineral base oils.
[35]	Pinilla-Peñalver et al., 2024	A new method for synthesizing polyurethane-based aerogels is proposed.

**Table 2:** The viscosity changes of four different samples from −20 °C to 30 °C.

Samples	Temperature Range (°C)	Starting Viscosity (mPa·s)	Ending Viscosity (mPa·s)	Percentage (%)
Sample 1	−20~30	90,541	13,619	84.9
Sample 2		11,675	2676.4	77.1
Sample 3		40,928	5837.9	85.7
Sample 4		72,771	10,062	96.2

**Table 3:** The viscosity changes of four different samples from 30 °C to 80 °C.

Samples	Temperature Range (°C)	Starting Viscosity (mPa·s)	Ending Viscosity (mPa·s)	Percentage (%)
Sample 1	30~80	14,127	7329.2	48.1
Sample 2		20,674	4777.9	76.8
Sample 3		10,426	3646.8	65.1
Sample 4		10,038	5404.6	45.2

**Table 4:** Rheological fitting equations for four different samples at different temperatures.

Samples	Temperature/(°C)	Fitting Equations (τ)	Fit Factor/(R^2^)
Sample 1	−20	$\tau =1048.2+231.55(\dot{\gamma }{)}^{0.74}$	0.9998
	30	$\tau =528.38+14.16(\dot{\gamma }{)}^{0.76}$	0.9996
	80	$\tau =240.71+51.72(\dot{\gamma }{)}^{0.23}$	0.9814
Sample 2	−20	$\tau =2046.8+136.63(\dot{\gamma }{)}^{0.69}$	0.9512
	30	$\tau =337.08+46.221(\dot{\gamma }{)}^{0.69}$	0.9991
	80	$\tau =247.31+1.36(\dot{\gamma }{)}^{0.88}$	0.9898
Sample 3	−20	$\tau =28.94+715.24(\dot{\gamma }{)}^{0.47}$	0.9959
	30	$\tau =409.3+12.807(\dot{\gamma }{)}^{0.74}$	0.9997
	80	$\tau =153.88+6.82(\dot{\gamma }{)}^{0.59}$	0.9966
Sample 4	−20	$\tau =2042.4+0.3(\dot{\gamma }{)}^{1.17}$	0.9884
	30	$\tau =337.35+19.16(\dot{\gamma }{)}^{0.67}$	0.9995
	80	$\tau =127.36+2228.5(\dot{\gamma }{)}^{0.01}$	0.9659

## Data Availability

The data presented in this study are openly available in article.
